# National monitoring of iodine, sodium, and vitamin D status in toddlers and women of childbearing age – results and lessons learned from a pilot study in Norway

**DOI:** 10.29219/fnr.v67.9088

**Published:** 2023-08-31

**Authors:** Synne Groufh-Jacobsen, Marianne Hope Abel, Anne Lise Brantsæter, Maria Andersson, Haakon E. Meyer, Sigrun Henjum

**Affiliations:** 1 Department of Nutrition and Public Health, Faculty of Health and Sport Science, University of Agder, Kristiansand, Norway; 2 Department of Physical Health and Ageing, Norwegian Institute of Public Health, Oslo, Norway; 3 Department of Food Safety, Division of Climate and Environmental Health, Norwegian Institute of Public Health, Oslo, Norway; 4 Nutrition Research Unit, University Children’s Hospital Zurich, Zurich, Switzerland; 5 Department of Nursing and Health Promotion, Faculty of Health Science, Oslo Metropolitan University, Oslo, Norway

**Keywords:** iodine, iodine status, iodine fortification, national monitoring program, thyroglobulin, salt intake, salt excretion, child health, Norway, vitamin D

## Abstract

**Background:**

Norway is lacking a population-based national monitoring program for iodine, sodium, and vitamin D status.

**Objective:**

The aim of this study was to pilot-test a study design for collecting biological samples from a country-representative sample of 2-year-old children and their mothers and to report results for iodine, salt, and vitamin D at baseline, before initiation of salt iodization in Norway.

**Design:**

In a cross-sectional study, we recruited 2-year-old children and their mothers during the routine 2-year check-up through 38 randomly selected health clinics in 2021. Spot urine samples were analyzed for iodine, creatinine, and sodium, and dried blood spots from the mothers were analyzed for thyroglobulin (Tg) and 25-hydroxyvitamin D (25(OH)D).

**Results:**

We aimed at including 400 mother–child pairs but recruited only 55 pairs. Major challenges were closed health clinics due to the COVID-19 pandemic, lack of motivation of the health personnel to prioritize recruiting, missing information about non-participation, and high workload for participants. The median urinary iodine concentration (UIC) was 123 (95% CI: 76, 228) µg/L in the toddlers and 83 (95% CI: 72, 99) µg/L in the mothers. The median urinary sodium concentration (UNaC) was 62 (95% CI: 37, 91) mmol/L in the toddlers and 93 (95% CI: 77, 107) mmol/L in the mothers. Of the mothers, 18% had levels of 25(OH)D <50 nmol/L (suboptimal status).

**Discussion and conclusion:**

Lessons learned from the pilot study will be used to design a national monitoring program for toddlers and women of childbearing age in Norway. The results indicate that 2-year-old children and women of childbearing age in Norway may have inadequate iodine intakes at the group level, while for vitamin D, most of the mothers had adequate status.

## Popular scientific summary

- Norway lacks a national monitoring program for iodine, sodium, and vitamin D status.
- We aimed to collect representative information from 400 toddlers and their mothers using a randomized cluster design.
- Recruitment through public child health clinics was challenging.
- Recruitment obstacles included high workload and pandemic lockdowns among health clinics, high participant burden, and lack of possibility for direct contact between project workers and participants.

National monitoring of nutritional status is important to provide country-representative data before implementing a nutrition intervention strategy and to evaluate the impact of an intervention program (1). Routine national monitoring of iodine, sodium, and vitamin D is recommended by the World Health Organization (WHO) (2) and implemented in many countries worldwide (3). However, there is currently no monitoring program established for any of these nutrients in Norway.

National monitoring of nutrient intake in the population is mainly performed using dietary assessment and evaluation of data on food sales. However, for some nutrients, data on food consumption are not reliable for the assessment of nutrient adequacy, and biological samples are needed to provide a more accurate estimate of nutrient status. For example, sun exposure is an important determinant of vitamin D status, and the blood concentration of 25-hydroxyvitamin D (25(OH)D) provides a valid measure of vitamin D status and captures both cutaneous vitamin D production and vitamin D from the diet and supplements (4). Vitamin D status is low in population groups that are less exposed to sunlight and do not take vitamin D supplements (4, 5). The vitamin D intake from foods is below recommended intake in the general population in all Nordic and Baltic countries, except for Finland (6).

Moderate to severe iodine deficiency was prevalent in Norway before the introduction of iodine fortification of animal fodder in the 1950s, which improved the iodine status in the Norwegian population through an increased iodine concentration in the cow’s milk. In Norway today, iodine fortification of the household salt is voluntary, the permitted level is 5 µg/g, and the main dietary iodine sources are lean fish, dairy products, and eggs (7, 8). Individuals with low consumption of these food groups are at risk of iodine deficiency if they do not use iodine-containing supplements. Cross-sectional studies on non-representative spot urine samples of different population groups in Norway have shown that iodine status is low in several population groups (9–13). Today, there is a need to increase the iodine intake in women of childbearing age in Norway, but at the same time, it is important to ensure that infants and young children will not be exposed to iodine excess (14).

The iodine content in different foods and supplements varies (11), and in countries with iodized salt, the information about the iodine concentration in industrial food products is limited. Thus, calculating iodine intake based on intake data is uncertain (15). The median urinary iodine concentration (UIC) is a well-established measure of iodine status at a group level (2, 15–18). The habitual iodine intake can also be estimated by accounting for urine volume by measuring the urinary creatinine concentration and by correcting for intraindividual variability by collecting two spot urine samples from non-consecutive days in a subsample of participants (3). Based on this, the prevalence of inadequacy or excess may be estimated (15).

A previous national dietary survey suggests that the salt intake in Norway is twice the recommended intake (19); however, salt intake is difficult to estimate from dietary surveys. Urinary sodium measured in 24 h urine or spot urine samples can be used as a population indicator for sodium intake (20, 21).

Today, salt iodization is the recommended strategy to increase population iodine intake (2), and it is currently being considered by the Norwegian health authorities. Therefore, it is important to establish a routine national monitoring program for iodine, in which women of childbearing age and young children are prioritized, as low iodine intake has been identified in women of childbearing age (11). An increased iodization level of salt and/or bread could potentially impose iodine intakes above upper limit for young children (14). Monitoring of iodine and salt in the same program gives added value since the contribution of salt to the total iodine intake can be estimated (22).

The aim of this pilot study was to test a design for collecting biological samples from a country-representative sample of 2-year-old children and their mothers and to report results for iodine, salt, and vitamin D at baseline, before initiation of salt iodization in Norway.

## Methods

### Subjects and study design

We aimed to include a representative national sample of 400 toddlers (2 years old) and their mothers in this observational cross-sectional pilot study conducted between January and October 2021. In a two-step cluster sampling procedure, municipalities were randomly selected, and the probability of selection of a municipality depended on the number of children born in each municipality in the first quarter of 2019, using available data from Statistics Norway. The selection of municipalities was stratified by country region (two from Northern Norway, three from Mid Norway, four from West Norway, and 11 from the South-East Norway regions). Health clinics were then randomly selected within the municipalities from a list of all health clinics in the selected municipalities. Each health clinic was asked to invite up to 30 mother–child pairs to participate in the study. Small health clinics were asked to recruit fewer, and then health clinics nearby were included to reach the goal of 30 invited mother–child pairs from that site.

The public health nurses at the selected health clinics were asked to invite all children scheduled for a 2-year checkup. The public health nurses were instructed to register the number of participants asked to participate, the number of participants who declined to participate, and the number who accepted to participate. The public health nurses were also instructed to provide brief information about the study to all potential participants. All mothers attending the 2-year-old check-up at the respective clinics and who could read and write Norwegian were considered eligible for inclusion. If the children were twins or triplets, one of them was invited to participate.

Mothers who agreed to participate received pre-packed bags with study information, links to online instructional videos and a web-based questionnaire, and written information on how to collect, store, and return the biological samples (urine and blood), along with necessary equipment. The participants had the opportunity to contact the research staff during the study by phone or email, but the research staff did not have information about individual participants and could not contact any participants when the study was ongoing.

The mothers were asked to complete a short web-based questionnaire that assessed background characteristics (20 questions) and dietary data for both the mother and the 2-year-old child (47 questions), for more information, see Supplementary Table 1. The questionnaire measured the habitual frequency consumption of selected food groups for iodine, salt intake, and vitamin D in the mother–child pairs. Based on the responses in the web-based questionnaire for supplement use in the previous 4 weeks (ranging from never to daily), the mother–child pairs were categorized as supplement users and non-supplement users separately for selected supplements (cod liver oil, omega-3 fatty acids, vitamin D, iodine, multivitamin, macroalgae, and multivitamins). If reporting using an iodine containing supplement or use of macroalgae occasionally or daily, they were categorized as iodine-supplement users. Likewise, if reporting using a vitamin D containing supplement occasionally or daily, they were categorized as vitamin D-supplement users.

### Analysis of iodine, creatinine, and sodium concentration in the urine and analysis of thyroglobulin (Tg) and vitamin D (25-OH-D) in dried blood samples

The mothers were instructed to collect one spot urine sample (5 mL) from the child, using Sterisets Urine collection pack 310,019 (SteriSets International BV, Oss the Netherlands) containing one syringe (5 mL), one specimen container (20 mL), and two urine collection pads (21 cm × 7 cm). Each of the Steriset Urine collection packs contained two urine collection pads in case of contamination with feces. The mothers were also instructed to collect one spot urine sample at random times from themselves (not the first morning void) in a labeled 100 mL Vacuette urine breaker (Greiner Bio-One, Kremünster, Austria) and to withdraw a subsample of urine from the breaker into a 9.5 mL Vacuette Urine Tube (Greiner Bio-One, Kremsmünster, Austria). One in four mothers was asked to collect spot urine samples from two, non-consecutive days from herself and from the child and was provided with double sets of equipment for both the mother and the child. However, in this pilot study, only one of the spot urine samples was used.

UIC was measured at ETH Zurich/University Children’s Hospital Zurich (Zürich, Switzerland) using the Pino-modification of the Sandell-Kolthoff method (23). The laboratory participates successfully in the Program to Ensure the Quality of Urinary Iodine Procedures (US Centers for Disease Control and Prevention, Atlanta, GA), and the analysis was run along with quality control samples to ensure the accuracy of the analysis. Analyses of urinary sodium and creatinine were performed at Fürst Medical Laboratory, Oslo, Norway. For urinary sodium, the Siemens Advia Chemistry XPT system was used, in which natrium is analyzed using ione-selective electrodes (ISEs) (Germany). Dried blood spot samples (DBSs) were collected by a finger prick onto filter paper cards (IDBS-226, Perkin Elmer, CT, USA) using a disposable lancet. Thyroglobulin (Tg) was measured using a DBS-Tg sandwich enzyme-linked immunosorbent assay (ELISA) at ETH Zurich/University Children’s Hospital Zurich (Zürich, Switzerland). Serum control samples (Liquicheck Tumor Marker Control, Bio-Rad, Hercules, CA, USA) mixed with red blood cell concentrate (blood donation Swiss Red Cross, Zurich, Switzerland) were used as standards for the DBS-Tg assay (24). Quality control was ensured using in-house DBS-Tg control samples.

Analysis of vitamin D (25-OH-D) in DBS was performed at Vitas As, Oslo, Norway. For analysis of vitamin D from the DBS, punches of whole blood from the mothers were diluted with water (hypergrade for HPLC-MS). After whole blood dilution, the analyte was extracted with 2-propanol (hypergrade for HPLC-MS) containing internal standard. Followed by analyte extraction, the sample was centrifuged – for removal of whole blood debris – and the supernatant was transferred to a container applicable to the LC-MS instrument. The supernatant was injected into a Ultivo Tripple Quadruploe LC/MS (Agilent Technologies, Santa Clara, CA, USA), separated by a Kinetex^®^ 2.6 µm C18,100, LC column 100 × 4.6 mm (Phenomenex, Torrance, CA, USA), and quantified by APCI-MS/MS.

### Definitions

Iodine sufficiency was defined as median UIC ≥100 µg/L in the mothers and the 2-year-old children (2). Additionally, for the 2-year-old children, the proposed median UIC cut-off at ≥200 µg/L was used to indicate potential iodine sufficiency (25). Reference values for the DBS-Tg method are currently lacking for women of reproductive age but are available for pregnant women (0.3-43.5 µg/L) (26). In the absense of high accuracy data, the reference range for pregnant women was applied for the mothers in this study. For the evaluation of vitamin D, levels ≥50 nmol/L indicated sufficient status (27).

### Ethics

This study was approved by the Regional Committee for Medical and Health Research Ethics, 2020/184344/REC South-East, and the Norwegian Center for Research Data/NSD/NSD286210. The data collection and the study were performed according to the Helsinki declaration. Study consent was provided before participation. The mothers were also offered to receive results on their tests of thyroid function and vitamin D status.

### Statistics

The normality of the data was checked using a visual evaluation of the Q–Q plots and histogram. Normally distributed continuous variables are reported as mean ± standard deviation (SD) and non-normally distributed continuous variables as median and bootstrapped 95% confidence interval (CI) or with the interquartile range (IQR). The Mann–Whitney U test was used to explore the difference in UIC and Tg, by iodine supplement users and non-use. Independent samples T-test was used to investigate the difference in 25-(OH)-Vit D in vitamin D supplements users and non-users. *P*-values <0.05 was considered statistically significant for all tests. The software used for statistical analysis was IBM SPSS statistics versions 27 and 28 (IBM Corp., Armonk, NY, USA).

## Results

From January to October 2021, mother–child pairs were invited to participate in the study at the health clinics at the 2-year health check. In total, 48 health clinics were contacted by e-mail and telephone and invited to help recruit participants (South Norway, *n* = 23; West Norway, *n* = 11; Mid Norway, *n* = 8; and North Norway, *n* = 6), of which eight declined and two health clinics did not respond. Due to the COVID-19 pandemic, the recruitment of participants was low in several of the health clinics because of repeated lockdowns, and no physical visits to the health clinics were allowed during large parts of the period allocated for data collection.

By November 2021, 55 mother–child pairs were included in the study from 38 health clinics (South Norway, *n*= 18; West Norway, *n* = 11; Mid Norway, *n* = 5; North Norway, *n* = 6). The health clinics were then asked to stop recruitment. Important challenges, learnings from the pilot study, and suggested modifications to the protocol for monitoring are presented in Table 1.

**Table 1:** Major learnings from the pilot study and suggested modifications to the protocol for biomonitoring of nutrient status in 2-year-old children and women of childbearing age

Challenges	Probable causes and consequences	Suggested modifications to the protocol
Low participation	The COVID-19 pandemic caused repeated lockdowns in many health clinics and increased the workload on the health personnel. The study staff did not have direct contact with the study participants during lockdowns. Healthcare staff reported having limited time and motivation to inform potential participants about the study. We were only able to have personal communication with the managers of the health clinics (by e-mail and/or phone) and not directly with the health personnel. Invited participants received a bag with equipment for biological sampling and information, which might have been perceived as somewhat overwhelming. There was no reward for participation except personal results for blood tests (vitamin D and thyroid function).	Change the recruitment method from inviting through health clinics as recruiting through health clinics would require on-site follow-up study personnel, which is resource demanding. Participants will be recruited through the National Population Register, and digital post service will be used for sending out the study invitations. Limit the burden of participation. Drop self-administered blood sampling. Participants will receive a personal invitation to participate, and equipment will only be sent by post to participants who agree to provide urine spot samples in addition to answering the electronic questionnaire. Reward participation with a gift card.
No information on non-participants	It proved difficult/not possible to obtain data on the participation rate from the health clinics. The health clinics had been instructed to register the number of participants asked to participate, the number who declined, and the number who accepted (i.e. took the bag with them). However, in practice, these data were missing from most of the health clinics, and, thus, we do not know the number of participants invited.	Invite a randomly selected (defined) sample drawn from the National Population Register of Norway and record the response rate.
Second caregiver who came to the health clinic was not invited	Many children were accompanied to the clinic by their second caregiver. Spot urine samples needed to be collected from the birth mothers, and thus, only birth mothers were invited. This might have caused selection bias.	Use a digital post service to recruit participants, instead of recruiting via health clinics.
Missing consent from the second caregiver, and no way to remind them	For the child to participate in the study when providing biological samples (urine), we needed the consent of the second caregiver. The second caregiver had to be informed and ‘recruited’ by the birth mother. A separate consent form was completed by the second caregiver.	We will contact the father directly with a separate study invitation, and we will have the opportunity to send a reminder.
Analyses were performed batch-wise, which caused a large delay from the test day to the results being available	Feedback to the participants concerning vitamin D status and thyroid function is of limited value when provided with a long-time lag.	As dried blood spot data will not be collected, individual feedback is not relevant.

The background characteristics of 55 mother–child pairs are presented in Table 2. The sample consisted mostly of women reporting more than 16 years of education (80%), no-smoking (100%), and with a mean age of 35 ± 6 years. In the sample, neither the infants nor the mothers reported having a vegan or vegetarian diet. However, nearly one-tenth of the mothers reported that both she and the child had a flexitarian diet, defined as reduced consumption of meat/meat products. Nearly one-fifth of the women were either pregnant, lactating, or planning a new pregnancy. Use of supplements was reported by three-quarters of the mothers; however, the type of supplements and frequency of use varied. Less than one-tenth of the mothers reported iodine supplement use within the last month, and one-quarter reported vitamin D supplement use. For the 2-year-old children, one-tenth were still breastfed daily, and three-quarter were provided with a dietary supplement (vitamin D, multivitamin/mineral, and omega-3).

**Table 2:** Background characteristics of Norwegian mother–child pairs (n = 55).

Characteristics	Children, *n* = 55
Gender Girls, *n* (%) Boys, *n* (%)	24 (44) 31 (56)
Age, months^a^	27 ± 4
Height, cm^a^	91 ± 13
Weight, kg^a^	13 ± 2
Age by weight measurement (in months)^a^	26 ± 4
Breastfeeding status, occasionally, *n* (%) Breastfeeding, daily, *n* (%)	2 (4) 6 (11)
Food allergy/intolerances, *n* (%)	1 (2)
Supplement use (all supplements) previous month, *n* (%)^b^	47 (86)
Iodine-containing dietary supplements previous month, *n* (%)	0
Macroalgae consumption previous month, *n* (%)	1 (2)
Flexitarian dietary practice, *n* (%) Vegan/vegetarian dietary practice, *n* (%)	4 (7) 0
	Mothers, *n* = 55
Age, years^a^	35 ± 6
Body mass index, kg/m^2^^a^	24 ± 4
Pregnant, *n* (%)	9 (16)
Lactating, *n* (%)	11 (20)
Planning pregnancy, *n* (%)	7 (13)
≤12 years of education, *n* (%) 12–15 years of education, *n* (%) 16 years of education, *n* (%)	1 (2) 10 (18) 44 (80)
Smokers, *n* (%)^c^	0
Snuff use, *n* (%)^c^	8 (15)
Food allergy/intolerances, *n* (%)	6 (11)
Flexitarian dietary practice, *n* (%) Vegan/vegetarian dietary practice, *n* (%)	5 (9) 0
Use of medication for a thyroid disorder, *n* (%)	3 (6)
Dietary supplement use (all types), *n* (%)^e^	40 (73)
Iodine-containing dietary supplements previous month, *n* (%)	10 (18)
Macroalgae consumption previous month, *n* (%)	0
Vitamin D, supplement use previous month, *n* (%)	14 (26)
Multivitamin (containing iodine), supplement use previous month, *n* (%)	8 (15)

a Presented as mean ± SD;

b includes cod liver oil, omega-3 fatty acids, vitamin D, iodine, multivitamin, and macroalgae;

c includes occasionally and daily use;

e includes cod liver oil, multivitamins, vitamin D, iodine, folate, and macroalgae.

### Iodine status in the children and the mothers

For the 2-year-old children, the median UIC was 123 µg/L (bootstrapped 95% CI: 76, 228) (*n* = 41) (Table 3**)**. For the mothers, the median UIC was 83 (95% CI: 72, 99) µg/L (Table 3), and 12% had a median maternal UIC < 50 µg/L. Median maternal Tg was 24 (95% CI: 19, 29) µg/L (range: Min‒Max, 2‒68 µg/L), and there was no correlation between maternal UIC and maternal Tg (*r*_s_ = 0.016, *P* = 0.931) or between iodine supplement use and Tg (*r*_s_ = -0.142, *P* = 0.425).

**Table 3:** Urinary iodine concentration, thyroglobulin, urinary concentration of sodium, urinary creatinine concentration, and 25(OH)D in mother–child pairs

	Children				Mother			
	Median	IQR	(95% confidence interval)	n^a^	Median	IQR	(95% confidence interval)	n^a^
UIC, µg/L^b^	123	178	(76, 228)	41	83	61	(72, 99)	43
Tg, µg/L^c^	–	–	–	–	24	18	(19, 29)	34
UNaC, mmol/L^d^	62	66	(37, 91)	34	93	50	(77, 107)	43
UCC, mmol/L^e^	2	3	(2, 3)	34	9	7	(7, 12)	43
					Mean	SD	(95% confidence interval)	*n*
25(OH)D, nmol/L^f^	–	–	–	–	67	22	(57, 76)	41

a Number of participants;

b UIC = urinary iodine concentration;

c Tg = thyroglobulin;

d UNaC = urinary natrium concentration;

e UCC = urinary creatinine concentration;

f 25(OH)D, nmol/L is presented as mean ± SD for the mothers.

### Sodium concentration and estimated excretion in the mother–child pairs

The median urinary sodium concentration (UNaC) was 62 (95% CI: 37, 91) mmol/L in the children and 93 (95% CI: 77, 107) mmol/L in the mothers (Table 3).

### Vitamin D status in the mothers

The mean ± SD concentration of 25(OH)D was 67 ± 22 nmol/L (95% CI: 57, 76) (range: min‒max, 31‒105 nmol/L) (*n* = 41), and 18% had 25(OH)D concentrations <50 nmol/L (Table 3). Mothers consuming vitamin-D-containing supplements (*n* = 24) had higher 25(OH)D nmol/L than those who did not (*n* = 17), 73 ± 22 compared to 59 ± 19, respectively, *P* = 0.035.

## Discussion

In this pilot study, we aimed to test a design for collecting biological samples from a country-representative sample of 2-year-old children and their mothers and to report results for iodine, salt, and vitamin D at baseline, before initiation of salt iodization in Norway. This study was designed as a cluster-based study (2). However, the response rate was much lower than anticipated, and the study results are affected by selection bias and can therefore not be considered nationally representative. Recruitment through the health clinics was challenging, not only because of the COVID-19 pandemic but also because the health personnel at the health clinics had a high workload and lacked motivation for recruitment. Due to a limited budget, this study personnel could not visit the invited health clinics or participate locally in the recruitment of participants. We did not have access to the names or personal information and could not contact invited participants, while the recruitment was ongoing, which also resulted in no information on the participation rate. In future studies, we suggest inviting a randomly selected (defined) sample drawn from the National Population Register of Norway and record the response rate.

The learnings from this pilot study have provided us with valuable information on how to reduce the burden on the participants in future monitoring. Currently, a second pilot study has been initiated based on the learnings from this present study, in which mothers are invited to participate first by completing the electronic questionnaire with the background demographics and the dietary questions for the mother and child. Thereafter, the mothers are asked if they want to donate a spot urine sample from herself and the child. All participants who donate urine samples receive a gift card of 500 NOK. A randomized subsample of participants (25%) is invited to donate urine samples from two non-consecutive days.

Adequate iodine nutrition is especially crucial during early childhood and in women of childbearing age. In this pilot study, the median UIC in the 2-year-old children was 123 (95% CI: 76, 228) µg/L, indicating iodine sufficiency according to the cut-off at 100 µg/L by WHO. However, the 95% CI indicates uncertainties in the estimates as they overlap with the established cut-off value at 100 µg/L. For the 2 year olds, our results are in line with findings in a previous study from 2013 to 2014, which included 18 months old children (*n* = 416) recruited in nine health clinics from around the country (28), and they reported a median (25th,75th) UIC of 129 (81, 190) µg/L. In countries with successful salt iodization, the reported median UIC in infants and toddlers ranges from 230 to 350 µg/L (25, 29).

For the mothers, the median UIC was 83 (95% CI: 72, 99) µg/L, both the group median and the 95% CI are below the threshold of 100 µg/L defined by WHO (2), confirming iodine insufficiency in women of childbearing age reported in previous studies (10, 11, 13). This population group has also repeatedly been found to have inadequate intake not only in Norway but also elsewhere in Europe (3). A previous study found that iodine supplement use is a predictor of UIC in women of childbearing age in Norway (11), but the sample size in our study was too small to evaluate the effect of iodine supplementation.

According to a recent benefit and risk assessment on iodine fortification of table salt and salt used in bread and other bakery products in Norway, fortification with 15‒20 mg iodine per kg salt will likely ensure sufficient iodine intake in women of childbearing age and other groups with low iodine intake but, may at the same time, increase the risk of 1- and 2-year-old children having iodine intakes above the upper safe level (13, 14). However, the potential risk was estimated based on dietary survey data, using food frequency questionnaires (FFQ), and not on UIC measurements (13). The UIC measurements in a previous study indicated that iodine intake calculated based on the FFQ in the benefit and risk assessment was overestimated, and that the risk of excess is low (28).

Tg has been suggested as a potential biomarker for the evaluation of iodine status (15); however, there are no current available reference values for the general population for the DBS-Tg method used in this study. If using the established reference values for pregnant women (0.3-43.5 µg/L) (26), in our study, 12% had Tg values above 43.5 µ/L. In previous studies, mild iodine deficiency has been associated with elevated Tg in pregnant women (26).

The Norwegian health authorities have several initiatives aimed at reducing salt intake. Food producers have been engaged in a so-called ‘salt partnership’, a collaborative project established to reduce salt intake in food products available for Norwegian consumers (30). In our study, the median UNaC in women was 93 (95% CI: 77, 107) mmol/L. This agrees with what was reported in (*n* = 243) women, aged 40‒69, who participated in the Tromsø Study in 2015‒16 where the mean spot UNaC was 86 mmol/L (31). In the Tromsø study, daily median salt intake in the same women was estimated to be 7.6 g/day based on a 24-h urine collection (31).

In our study, 18% of the women had 25-(OH)-VitD <50 nmol/L, indicating a low prevalence of suboptimal vitamin D (27). A previous study found that intake of vitamin D from diet and supplements was associated with sufficient levels of 25-(OH)-VitD [34]. Vitamin D supplement users in our study had statistically higher 25-(OH)-VitD than non-users, supporting the general advice to use a vitamin D supplement to achieve the recommended intake of 10 µg/day if dietary intake is low, and there is little cutaneous vitamin D production (winter).

### Strengths and limitations

At the population level, spot urine samples can reflect the distribution of habitual intake of iodine if one collects repeated spot urine samples from a subsample of participants (15). In this pilot study, there are not sufficient data to identify groups at high risk of too low or too high intakes due the small sample size and large uncertainty in the data. However, the median concentration of iodine will indicate status at a group level. If urinary potassium and creatinine had been analyzed in the spot urine samples, 24-h urinary excretion of sodium could also have been calculated (31).

Most of the participating mothers were highly educated and non-smokers, suggesting that the study attracted participants with higher socioeconomic status compared to the general population. There were missing data on non-participants.

A strength of this study was the use of biological samples as objective measures, which are not subject to self-reporting bias. We also collected information about the intake of selected foods and the use of dietary supplements, which is valuable for exploring determinants of intake and for comparison of results over time with future data collections. However, there are measurement errors related to the sampling and the analysis. All mothers were provided equal instructions on how to collect and store all the samples, which is a strength. Assessment of 25(OH)D using DBS has been validated with the cut-offs applied in this study; however, for accurate measures, trained people in obtaining DBS samples is needed (32). In future monitoring, we suggest omitting blood sampling to reduce the burden on the participants. Thus, continued effort in the establishment of a routine monitoring program for vitamin D status is still needed.

## Conclusion

Norway is lacking a national monitoring program for iodine intake, sodium intake, and vitamin D status. Lessons learned from this pilot study with regard to study design, recruitment of participants, and participation burden will be used in drafting a routine monitoring program for iodine, sodium, and potentially other nutrients and environmental contaminants in toddlers and women of childbearing age. Major adjustments to the protocol are suggested for future monitoring studies. Results from this pilot study agree with previous findings, indicating that women of childbearing age are mildly iodine deficient, have a salt intake higher than recommended, and most have a sufficient vitamin D status. The data also suggest toddlers in Norway may have inadequate iodine intake. Data at the national level in a larger study sample are required to provide more reliable and precise estimates and to estimate subgroups at risk of too high or too low intakes. In a follow-up study, we recommend that the sample should be drawn from the National Population Register, and that participants should be recruited through direct invitation by mail.

## Data Availability

The data will be provided upon reasonable request.
